# Population Genetic Structure and Demography of the Critically Endangered Chequered Blue Butterfly (*Scolitantides orion*) in a Highly Isolated Part of Its Distribution Range

**DOI:** 10.3390/insects11090608

**Published:** 2020-09-08

**Authors:** Magdalena Czajkowska, Łukasz Dawidowicz, Anetta Borkowska, Izabela Dziekańska, Marcin Sielezniew

**Affiliations:** 1 Department of Zoology and Genetics, Faculty of Biology, University of Bialystok, Ciołkowskiego 1J, 15-245 Białystok, Poland; abork@uwb.edu.pl (A.B.); i.dziekanska@uwb.edu.pl (I.D.); marcins@uwb.edu.pl (M.S.); 2 Department of Zoology, Faculty of Biology and Biotechnology, Maria Curie-Skłodowska University, Akademicka 19, 20-033 Lublin, Poland; mori666@o2.pl

**Keywords:** butterfly conservation, genetic variation, microsatellites, mtDNA, population ecology, population genetics, *Scolitantides orion*

## Abstract

**Simple Summary:**

The disappearance of many butterfly species is currently observed in Europe, as most of them display strict habitat preferences and/or food specializations. A good example of such a species is the chequered blue butterfly (*Scolitantides orion*), whose caterpillars feed only on a few species of sedum and are guarded by ants. In Poland, this butterfly has survived only in one region and it is critically endangered. It is important to examine the genetic condition and demography of its extant populations, to help to create an effective conservation plan for the species. We studied the demography and genetic structure of the two largest populations inhabiting opposite banks of the Vistula River. Captured individuals were marked and released to estimate population sizes. Both populations were small, and they fluctuated in numbers, but adults were twice as numerous on the western riverbank. Analyses of various genetic markers have shown that the genetic variation is low in each population. Likewise, the genetic diversity between the two populations is low, which indicates that the gene flow exists, despite the river acting as a geographical barrier. The occasional migration occurs more frequently from the west to the east, which is consistent with the dominant regional wind direction.

**Abstract:**

*Scolitantides orion* is a butterfly species threatened in many European countries. In Poland, it survived in a single highly isolated area (Vistula River valley), which is an example of the dramatic decline in the population number. We studied the two largest remaining populations inhabiting opposite banks of the river. Mark-release-recapture studies showed that both populations were small, and they fluctuated in numbers, but adult individuals were twice as numerous on the western site. Genetic analyses were carried out using a mitochondrial (COI, ND5) and nuclear markers (Wgl, EF-1α, and microsatellite loci). We found out that genetic variation was low at both sites but higher in the smaller eastern population. This pattern is likely to be better explained by past distribution, when the butterfly, as a continental species used to be much more widespread in the east. However, the genetic differentiation between populations was low. This could suggest that the existing gene flow is facilitated by dominant regional wind direction, which may also contribute to a better genetic condition of the western population. Finally, a comparison of the obtained COI sequences with others available enabled us to reveal the phylogeographic pattern of the *S. orion* from different localities within its range.

## 1. Introduction

Butterflies are considered to be sensitive to environmental changes [1]. The main reason for the disappearance of many butterfly species observed in Europe is the loss of natural habitats or their fragmentation, caused by the processes of natural succession and human activities. Climate changes may also contribute to local declines and range shifts [2]. Highly specialized species living in scattered and not very numerous populations are the most vulnerable [3]. Becoming more and more isolated, they are threatened by stochastic factors leading to extinctions or at least serious bottlenecks, which potentially reduce genetic diversity and therefore fitness [4,5,6,7].

It is suggested that as many as a quarter of European butterflies are threatened [8]. Moreover, the distribution is often very uneven and some species which are relatively widespread in some parts of its range could be critically endangered at a regional scale. A good example is the chequered blue butterfly *Scolitantides orion* (Pallas 1771) distributed irregularly across Palaearctic from Spain to Japan [9]. Its European range is disjunctive, the majority of the populations localized in the south and a few localities reaching Central Europe. The second part of its European range encompasses the southernmost part of Fennoscandia [10]. The species is classified as being of least concern on the European Red List [11], but in many countries, especially in the northern and central parts of the continent, it is threatened [12], and its occurrence is restricted to local isolated populations. In some countries, e.g., Finland, habitat restoration was undertaken to increase the population size of *S*. *orion* to a viable level [13].

Here, we investigated for the first time a genetic structure and demography of the chequered blue butterfly. Our studies were conducted in a highly isolated part of its distribution range in Poland. The two chosen local populations were the largest of all known in the country and coincidentally the only populations that were large enough to carry out thorough demographic and genetic research. Mark-release-recapture sampling (MRR) enabled us to estimate seasonal population size and the mean lifespan of adults. Then, using both mitochondrial and nuclear markers, we assessed genetic structure of the populations and examined the differentiation between them to detect possible gene flow. In this text, we also discuss implications of our findings for the regional viability assessment of the butterfly. In addition, based on a comparison of obtained sequences of the cytochrome oxidase subunit (COI) and others available in the GenBank database, we reveal the phylogeographic pattern of the *S*. *orion* from different localities within its range.

## 2. Materials and Methods

### 2.1. Study Species

The chequered blue butterfly *Scolitantides orion* (Lepidoptera, Lycaenidae) is one of the smallest European butterflies, with the wingspan of about 25–28 mm. Sexual dimorphism is moderately conspicuous. The upper side of females is very dark greyish-brown, with blue flush restricted to basal part or absent. In males, the blue markings often cover most of the wings, but their intensity can vary considerably. Six subspecies are recognized throughout its range, of which three occur in Europe, i.e., *S. o. orion* Pallas, 1771 (Central and SE Europe to Turkey and Western Siberia) (Supplementary Figure S1A–C), *S. o. parvula* de Saggara, 1926 (Iberian Peninsula and Pyrenees), and *S. o. ultraornata* Verity, 1937 (southern Fennoscandia) [14].

The butterfly inhabits warm, often rocky slopes scarcely covered by vegetation, with significant presence of larval food plants i.e., some *Sedum* species (mostly *S. album* and *S. telephium*). Local populations are usually small, but may form a metapopulation system [15]. There are 1–3 broods a year, depending on the locality and season [16]. Eggs are laid on leaves and caterpillars feed underside often guarded by ants from different genera. The species overwinters as a pupa [9].

In Poland *S. orion* was always a very local species, recorded in four areas. It became extinct in Lower Silesia, Pieniny Mountains and Bieszczady Mts., and has probably survived only in the valley of the Vistula River on a few sites in the area of Kazimierz Dolny [17]. In the 1990s the butterfly was also recorded in one more locality in the Vistula River valley and in the Polesia region in Eastern Poland [18], but it is commonly suspected that these observations resulted from unsuccessful introductions. The location of the butterfly nearest to the confirmed Polish populations is in Slovakia and it is situated about 250 km to the south [10].

### 2.2. Study Sites

During intensive exploration carried out between 2014 and 2016 in the area of Kazimierz Dolny only three local populations were found there. In the case of one of them, only few individuals were observed. Hence, this study was restricted to two sites about 9 km apart and separated by unfavourable biotopes, including: farmlands, village buildings, and the Vistula River about 300 m wide. They were Parchatka (P1) and Janowiec (P2).

The site of Parchatka (P1) is located on the eastern bank of the Vistula River in vicinity of Parchatka village and encompassed sunny edges of the ravine covered with patches of xerothermic vegetation, with a total area of about 0.13 ha (Supplementary Figure S2A). The ravine crossed cereal crops and raspberry plantations. Ecotones and mid-field balks were covered with segetal plants and perennials including invasive goldenrods (*Solidago canadensis*). A smaller patch of the habitat (a small clearing, 0.03 ha) was located at a distance of about 200 m. It covered the southern slope of the Vistula River valley. However, due to very low numbers of observed individuals, it was excluded from the analyses. In the first half of the 20th century, xerothermic grasslands and scrubs were much more prevalent in the area and there were likely a lot of interconnected patches of suitable habitats.

The site of Janowiec (P2) is situated at the edge of the Vistula River valley, on the west bank of the river, and encompassed about 2.5 ha of open sandy grasslands surrounded by the Rhamno-Prunetea scrub and low trees, mainly Scots pines *Pinus sylvestris* (Supplementary Figure S2B). The area was mostly flat with a small hill with gentle slopes. At a distance of about 300 m a smaller patch of habitat (ca 0.5 ha) of a different character was located. It included an enclave of xerothermic open vegetation on a steep slope (up to 45°) of the valley surrounded by dense scrub. Very few butterflies were recorded there, and demographic analyses were restricted to the larger patch.

### 2.3. Adult Demography

Both populations were sampled in 2015 and 2016 with the mark-release-recapture method (MRR). The phenology of the butterfly at both sites was similar and the main difference was the appearance of a few individuals of the second generation in the P2 population. The sampling covered the entire flight period of the focal species in the first brood. The P1 site was visited between 5 May and 11 June in 2015 and between 4 May and 1 June in 2016, 15 and 13 times respectively. In P2, the sampling was carried out between 30 April and 17 June in 2015 and between 29 April and 7 June in 2016. There were 15 visits in both seasons. One person spent about 2–3 h on the site during each sampling day and studies were performed on days with favourable weather, between 10 am and 5 pm. Butterflies were captured with an entomological net, marked on the underside of their hind-wings with unique identity codes using a fine-tipped waterproof pen, and then immediately released at the place of capture. Due to a small size and complicated wing pattern it was not possible to write a number as it is widely practiced in studies of other lycaenids, thus a combination of colour dots and dashes was applied (Supplementary Figure S1D,E). This marking system enabled us to identify every individual by sight when settled. Recapture and handling were necessary only in the case of specimens requiring closer inspection, i.e., those with heavily worn wings. The date and time of each (re)capture were recorded as well as the sex of every individual.

The data collected were analysed with the Cormack–Jolly–Seber and constrained Jolly–Seber models (POPAN), using MARK 8.0 software [19,20]. The models represent a well-established standard for estimating population size in open populations, and they have been frequently applied in butterfly studies [21,22,23,24]. Based on the lowest value of the Akaike information criterion corrected for small sample size (AIC_c_) [25], an appropriate model variant was applied. Models were used to obtain the estimates of numbers of males and females captured daily, and their seasonal population sizes. The latter were subsequently summed up to yield the overall population size. Finally, based on the survival estimates, we estimated the mean lifespan of adult butterflies as *ê* = (1 − ϕ)^−1^ − 0.5 [26] and temporal fragmentation index, i.e., the relationship between flight period and average lifespan [27].

### 2.4. Sampling for Genetic Studies and DNA Extraction

A total number of 65 individuals of *S. orion* was sampled in 2018 for genetic studies. Tissue samples were taken using a nonlethal method from 34 adults of *S. orion* from P1 population and 31 butterflies from P2 population. Small fragments of hind wings (~2 mm^2^) were torn off using tweezers for genetic testing, and then individually stored in 95% ethanol. All procedures were approved by the Regional Director for Environmental Protection in Lublin, Poland (permit no.: WPN.6401.57.2018.MPR). DNA was extracted from small wing clippings using the Genomic Mini Kit (A&A Biotechnology), following the manufacturer’s protocol, and eluted in 100 µL of Tris buffer.

### 2.5. Microsatellite Analysis

Samples were then genotyped at 12 highly variable microsatellite loci previously designed for the other butterfly species of the Lycaenidae and Nymphalidae families (Table 1). Out of these 12 microsatellite markers, 11 were organized into four multiplex PCR sets (tree, two, four and two loci, respectively) avoiding allele overlap between loci labelled with the same dye, and one locus was amplified separately. Multiplex PCRs were performed with Labcycler Gradient (SensoQuest GmbH, Goettingen, Germany) in 5 µL reaction volume containing 2 µL genomic DNA (~20 ng), 1.7 µL Qiagen Multiplex PCR MasterMix (1x), 0.3 µL mix of primers and 1 µL RNase-free water. Each multiplex PCR started with an initial activation step of 95 °C for 15 min, followed by 30/40/45 cycles (see Table 1) of denaturation at 94 °C for 30 s, annealing at 52 °C for 90 s and extension at 72 °C for 60 s, and ended with a final extension at 60 °C for 30 min. The PCR products were mixed with 10 µL ultragrade formamide and 0.2 µL GeneScan™500 LIZ size standard (Applied Biosystems, Foster City, CA, USA), denatured at 95 °C for 5 min, rapidly cooled and then subjected to fragment length analysis by using a four-capillary ABI 3130 Genetic Analyzer (Applied Biosystems, Foster City, CA, USA). The fragment lengths of microsatellite alleles were estimated automatically using the AutoBin feature in GeneMapper 4.0 software (Applied Biosystems, Foster City, CA, USA), and then checked manually. The software Micro-Checker 2.2.1. [28] was used for identifying possible genotyping errors (stuttering, large allele drop-out, false and null alleles frequencies) by performing 1000 randomizations. We also used Cervus 3.0.3 [29] to estimate the null allele frequency values (*F*null). Markers that showed high values of *F*null (Macu29, Macu40, Macari16, and Lb4/18) and monomorphic loci (Macu9 and Macu15) were excluded from further analysis (Table 1).

The remaining six loci were then analysed using the Cervus 3.0.3 program for determining the number of alleles per locus (A) and their size ranges, as well as to calculate the expected (H_E_) and observed (H_O_) heterozygosities, and the mean polymorphic information content (PIC). Linkage disequilibrium between loci was tested with Genepop v4.0 [36] using the Markov chain method (10 000 dememorization steps, 100 batches, 5000 iterations) and Fisher’s exact test. A Bonferroni correction for multiple testing was applied. Genepop v4.0. was also used for calculation of departures from Hardy–Weinberg equilibrium. The gene diversity per locus and population and the inbreeding coefficient *F*_IS_ in each population were calculated with Fstat 2.9.3 [37]. The genetic differentiation between the two populations of *S. orion* were estimated for six microsatellite loci by calculating the *F*_ST_ values, which quantify the variance of allele frequencies, in Fstat 2.9.3, as well as *R*_ST_ values using Genepop v4.0. The significance of *F*_ST_ value was ascertained with 1000 permutations and interpreted using Wright’s scale [38]. The 95% confidence interval (CI) was estimated in Fstat 2.9.3. We estimated gene flow directly by first generation migrant detection in GeneClass2 [39] to determine the probability of each individual originating in the population where it was sampled. The effective size of populations (*N*e) was estimated using the linkage disequilibrium method employed in LDNe [40]. Alleles with a frequency <0.01 were excluded from the LDNe analysis. Patterns of genetic differentiation were visualized by means of principal coordinate analysis (PCoA) based on Nei’s genetic distance matrices between individuals using GenAlEx6.41 [41,42].

### 2.6. PCR for Genes Amplification, Sequencing, and Genetic Diversity

We amplified and then sequenced fragments of two mtDNA genes (COI and ND5) and two nuclear genes (EF-1α and Wgl). Universal primers for barcoding gene COI, LepF1 and LepR1 [43] were used for the fragment gene amplification (648 bp) and the Wg1 and Wg2 [44] primers were used for amplification of the Wgl gene (393 bp). Primers for two remaining genes were designed using Primer3 (v 0.4.0) software [45]: Sor_ND5_F: CTTTAGTTACTGCTGGTG and Sor_ND5_R: ATTCTACCAGAAAAAACTC for ND5 fragment gene amplification (646 bp) and Sor_EF1α_F: GAAGTTCGAGACCGCAAAGT and Sor_EF1α_R: AAGAGCTTCGTGGTG CATCT for EF-1α gene (664 bp). PCRs for each primer pair were carried out in 5 µL volumes, and the reaction mixtures consisted of 2 µL of DNA, 1.7 µL of Qiagen Multiplex PCR Master Mix (1×), 0.3 µL of primer mixture (0.2 µM of each primer), and 1 µL od RNase-free water. The polymerase chain reaction cycling scheme was as follows: 15 min at 95 °C followed by 45 cycles of 30 s at 94 °C, 90 s at 52 °C (COI, Wgl), 55 °C (EF-1α) or 59 °C (ND5), 60 s at 72 °C and the final extension step of 30 min at 60 °C. PCR products were purified with the Clean-Up kit (A&A Biotechnology) and sequenced in both directions with the BigDye Terminator v3.1 Cycle Sequencing Kit (Applied Biosystems). The reaction conditions were as follows: 25 cycles with denaturation at 95 °C for 20 s, annealing at 50 °C for 15 s, extension at 76 °C for 60 s. Sequencing reaction products were purified with the ExTerminator kit (A&A Biotechnology, Gdynia, Poland) and separated on a 3130 Genetic Analyzer (Applied Biosystems, Foster City, CA, USA). The DNA Sequences were aligned in BioEdit v 7.0.4.1 [46] and revised manually for polymorphic site detection. Sequences of all haplotypes have been submitted to the GenBank database under accession numbers: MT820115, MT820116 (COI gene), MT820117, MT820118 (ND5 gene), MT820119-MT820121 (Wgl gene), and MT820122–MT820126 (EF-1α gene).

Haplotype reconstruction of the nuclear genes (EF-1α and Wgl) from genotype data was conducted using the algorithms provided in PHASE as implemented in DnaSP v.5.10 [47]. For all gene fragments, we calculated the number of haplotypes (Nh), haplotype diversity (h), nucleotide diversity (π) and number of segregation sites (S) using software packages Arlequin [48] and DnaSP v.5.10. Genetic differentiation between studied populations was assessed by pairwise *F*_ST_ and *Φ*_ST_ values. All values were statistically tested in Arlequin.

### 2.7. Phylogenetic Analysis

Due to small numbers of haplotypes identified in both Polish populations of the chequered blue butterfly and very few ND5, EF-1α and Wgl gene sequences available in the GenBank database, we conducted phylogenetic analysis only for the mtCOI gene. Phylogenetic analyses were performed on 93 sequences of the mtCOI gene fragment (579 bp), of which 64 were from Poland (P1: N = 34, P2: N = 30), and the other 29 sequences were from the GenBank database (Supplementary Table S1). Phylogenetic Bayesian coalescent reconstruction analysis of the subset of sequences consisting of distinct haplotypes of Polish populations and sequences available in the GenBank database as operational units was carried out in BEAST 1.75 [49]. The substitution model HKY  +  I (Hasegawa-Kishino-Yano, has Invariant sites) was used as suggested by jModelTest [50]. A total of 750 MCMC runs were performed, 5000 trees were saved and the first 25% discarded as burning. The convergence of MCMC runs and effective sample size (ESS  ≥  200) was assessed using Tracer v1.6 [51]. Trees were rooted using mtCOI sequences from the small blue butterfly (*Cupido minimus*, JQ996391) and the scarce large blue (*Phengaris teleius*, JQ996398) downloaded from GenBank. A consensus tree accessing the posteriori probability values of each clade was generated using TreeAnnotator 1.6.1 [49]. Haplotype network reconstruction was performed in Network v4.6.1.0 [52].

## 3. Results

### 3.1. Adult Demography

The number of marked individuals varied between 19 in 2015 (P1) and 74 in 2016 (P2, Table 2). Most of the marked individuals in both populations and in both seasons (61.2%) were observed only on the day of marking. A total number of 92 individuals (38.8%) was recorded on at least two different days.

The population size was significantly higher in 2016, both in P1 and P2 population. In three datasets, males were more numerous than females by about 30% and only for the P1 population predominance of females was detected in 2015. However, it is worth noting that the total number of marked individuals was in this case relatively low compared to the other datasets (Table 2).

The maximum duration between captures of an individual reached 17 and 11 days for males and females, respectively. The maximum number of recaptures on different days for an individual was 5 for males and 6 for females. The mean number of days between the first and last capture varied between 2.17 and 3.86 for males and 1.89 and 2.43 for females.

For two datasets (P1 from 2015 and P2 from 2016) the model assumed a constant (and equal for both sexes) survival rate (*φ*), while for the two other *φ* was sex-dependent and hence it was possible to calculate survival rates for both sexes separately. However, capture probabilities (*p*) were sex-dependent only for P1 from 2015 and P2 from 2016.

The daily survival rate varied between 0.73 for females in 2015 in P2 and 0.91 for males in 2016 in the P1 population, and the estimated adult lifespan varied between 3.17 and 10.68 days (Table 3). The mean calculated adult lifetime for both sexes was 6.55 days. Taking into consideration the mean recorded flight period for both populations in two seasons (38 days), the temporal fragmentation index was estimated at 5.80.

### 3.2. Genetic Variation at Microsatellites Loci, Gene Flow and Effective Population Size

The number of alleles per locus (A) ranged from 4 (Macu44) to 12 alleles (Macu5 and Malc169), on average N_A_ = 7.83 alleles for the total sample (N = 65) and six microsatellite loci (Table 4). The mean number of alleles per locus (N_A_) was low and very similar in both examined populations, at 5.83 (P1) and 5.67 (P2), respectively. The parameters of genetic variation, i.e., expected heterozygosity (H_E_), observed heterozygosity (H_O_), and the mean polymorphic information content (PIC) estimated on the basis of microsatellite loci, were similarly low in both populations, although slightly higher in the P1 population (Table 4). The gene diversity at the studied loci ranged from 0.142 to 0.685 in the P1 population and from 0.064 to 0.637 in the P2 population. *F*_IS_ values were negative and not significantly different from zero in both *S. orion* populations (*F*_IS_ = −0.134 and −0.054 in the P1 and P2 population, respectively). There was no significant linkage disequilibrium among any pair of the loci under study, indicating that the studied loci were most probably not linked. Deviations from Hardy–Weinberg equilibrium were significant after Bonferroni correction for two loci (boleun01 and Malc169), but only in the P1 population (Table 4).

GeneClass2 identified eight individuals (23.5%) from the P1 population as being possible first-generation migrants, and there was a significant probability that six individuals (19.4%) did not originate from the P2 population in which they were sampled. For the P1 population, LDNe revealed an *N*e of 29.4, with 95% parametric confidence intervals (CI): 16.0–70.8, whereas for the P2 population, the *N*e was 41.6 with a large 95% parametric CI: 19.5–209.8.

### 3.3. Sequence Polymorphism in Genes

We discovered only two distinct haplotypes for mtCOI gene, of which the one appearing in P1 population was new and not previously reported (Supplementary Table S1). The haplotypes were separated by four polymorphic sites (S). Gene diversity (h) was calculated to be 0.166 ± 0.080 SD, and nucleotide diversity (π) was 0.001 ± 0.001 SD in the P1 population. Similarly, low variability was noted for the mitochondrial ND5 gene with only two haplotypes, the same in both populations and previously unreported, separated by one polymorphic site (S). We also found new haplotypes among nuclear gene sequences from the studied Polish populations. Three haplotypes were identified in the Wgl gene (H1-H3 in P1, and H1 and H2 in P2), of which only one (H1) was previously described in an individual from Denmark (GenBank accession no. HQ918021). There were two polymorphic sites in this gene, one of which was common in both populations, and the other occurred in only one sequence in the P1 population. The highest number of haplotypes (5) was identified in sequences of the EF-1α gene (H1-H4 in P1, and H1 and H5 in P2). One of them (H1) was found in the GenBank database (accession no. AY675394), while the other four were not reported previously.

The parameters of genetic variation in all sequenced genes in both populations, are summarized in Table 5. The genetic variability estimated on the basis of gene sequence variability in both studied populations from Poland varied depending on the marker used. However, for the concatenated mitochondrial genes (COI_ND5) and the combined nuclear genes (Wgl_EF-1α), the gene diversity was greater in the P2 than the P1 population (Table 5).

### 3.4. Genetic Differentiation between S. orion Populations from Poland

According to Wright’s scale [38], pairwise genetic differentiation values for microsatellite loci between the two largest *S. orion* populations in Poland were low but significantly different from zero (*F*_ST_ = 0.021, 95% CI: 0.008–0.030; *p* < 0.001 and *R*_ST_ = 0.013, *p* < 0.05). In agreement with these results, the individual microsatellite genotypes of *S*. *orion* from both Polish populations showed overlapping distributions in the PCoA plot and did not show any obvious subgroups (Supplementary Figure S3). Similar to microsatellite loci, nuclear genes pairwise genetic differentiation values between the studied populations were low for the Wgl gene (*F*_ST_ = 0.035, *p* > 0.05 and *Φ*_ST_ = 0.043, *p* < 0.05); and moderate for the EF-1α gene (*F*_ST_ = 0.066 and *Φ*_ST_ = 0.085, and significantly different from zero: *p* < 0.05). Low values of *F*_ST_ = 0.013 (*p* > 0.05) and *Φ*_ST_ = 0.051 (*p* < 0.05) were also obtained for the concatenated nuclear gene sequences, EF-1α_Wgl. Low and not significantly different from zero values of *F*_ST_ and *Φ*_ST_ = 0.054 (*p* > 0.05) were observed also in mtCOI gene. Only in the mitochondrial ND5 gene genetic differentiation values were very great (*F*_ST_ and *Φ*_ST_ = 0.584) and significantly different from zero (*p* < 0.05), which translated into equally high values of these parameters obtained for the concatenated COI and ND5 gene sequences (*F*_ST_ = 0.510, *Φ*_ST_ = 0.426, *p* < 0.05). However, it should be emphasized that the high genetic diversity values derived from the ND5 gene sequence were the result of just one variable site in this gene fragment that differentiated the two populations.

### 3.5. Phylogeographic Pattern Inferred from COI Sequences

**Figure 1:**
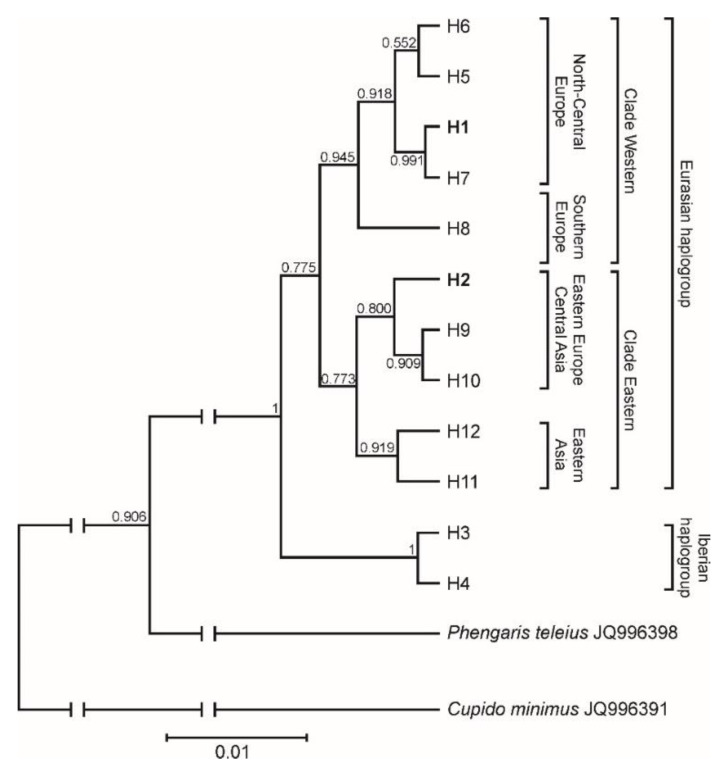
Phylogenetic Bayesian coalescent reconstruction analysis of the subset of mtCOI sequences consisting of distinct haplotypes noted in Polish populations (H1, H2) of *Scolitantides orion*, as well as sequences available in the GenBank database (H1, H3-H12). The tree has been rooted with sequences of the small blue (*Cupido minimus*, JQ996391) and the scarce large blue (*Phengaris teleius*, JQ996398).

**Figure 2:**
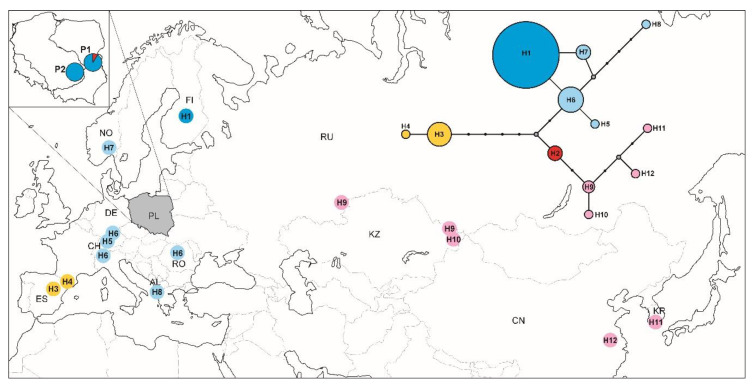
Distribution of COI haplotypes among populations of *Scolitantides orion* in Poland (PL) and different localities within its range (sequences available in the GenBank database). Haplotype network reconstruction was drawn using the median-joining method. The sizes of the circles on the haplotype network are directly proportional to the number of individuals analysed.

Phylogenetic analyses performed on 93 sequences of the mtCOI gene fragment in DnaSP v.5.10 and Arlequin programs have identified 12 haplotypes (see haplotype frequencies in Supplementary Table S1). The analysed individuals from both populations in Poland had the same haplotype (H1), which has so far been reported only in Finland (GenBank accession no. KT782365). However, three individuals from the P1 population had a new haplotype (H2) not previously described, different from the H1 haplotype by four polymorphic sites. Phylogenetic Bayesian coalescent reconstruction analysis of distinct haplotypes clearly distinguished two haplogroups of the chequered blue butterfly: Iberian and Eurasian haplogroups with high bootstrap support on phylogenetic trees (Figure 1). In the Eurasian haplogroup, two clades can be distinguished: Western and Eastern. Moreover, each of these clades can be divided into two groups: North-Central Europe and Southern Europe in the Western clade, and Eastern Europe-Central Asia and Eastern Asia in the Eastern clade. Almost all Polish individuals of *S. orion* possessing the H1 haplotype (Supplementary Table S1) grouped on the tree in the Clade Western and the North-Central Europe group next to the haplotypes recorded in individuals from Norway, Germany, Switzerland, and Romania. The remaining three individuals from the P1 population, characterized by H2 haplotype, which occurred on a tree in the Eastern Europe-Central Asia group of Clade Eastern, next to haplotypes from eastern Russia and Kazakhstan (Figure 1). A network was also drawn using the median-joining method to confirm possible relationships between mtCOI haplotypes found in this study and other COI mtDNA sequences downloaded from GenBank (Figure 2).

## 4. Discussion

### 4.1. Adult Demography

Low density populations are typical for *Scolitantides orion* and they are reported in both the northern and the southern parts of its European distribution range [53,54,55,56]. At the same time, small local populations are able to thrive for a long time. This study is the first attempt to estimate precisely local population sizes using the MRR method. Furthermore, *S. orion* showed to be a relatively long-lived butterfly, especially when compared to the other members of the Lycaenidae family. For example, the residency time of the closely related *Pseudophilotes bavius* was estimated at only 2.8 days [57]. Ref. [27] suggest that short-lived species could be vulnerable especially if they are characterized by higher values of the temporal fragmentation index. The value estimated in this study was relatively low and was in line with values recorded for species of least conservation concern. This positive trait may contribute to persistence of small populations, making finding a mate more likely [58].

A similar advantage could result from a longer lifespan of females, which could be suggested by some of our results. It is also important to note that it is not possible to distinguish emigration from mortality. Therefore, shorter residence time of females could be in fact related to differences in mobility of both sexes. If this is true, it would be consistent with studies performed in Finland, which showed that 71% of males and only half of females remained in a single patch [53]. After laying eggs, females may leave their habitat looking for other suitable patches. This kind of behaviour can be seen as a “spread-the-risk” strategy [59].

### 4.2. Genetic Diversity and Differentiation between Populations

Analyses revealed that genetic variation was low in both of the studied populations, although higher for the smaller eastern population. This pattern could be better explained by past distribution. The species was widespread in the suitable habitats on the eastern bank of the river as early as in the 1990s [17] and the total population could be probably counted in thousands of individuals. The Parchatka population (P1) was very likely a part of a metapopulation system, but has now significantly decreased. On the other hand, the Janowiec population (P2) is the only known in the west, although presently covering a much larger area and inhabited by at least twice as numerous a population.

Comparisons with studies on different species and using different microsatellite loci can be problematic. Nevertheless, it is worth noting that indices of genetic diversity were low but not extremely low when compared with other lycaenids, especially the most extensively studied *Phengaris* butterflies [60,61,62,63,64]. Therefore, although our ecological studies indicate some fluctuations in population sizes, genetic data did not indicate drastically reduced genetic variation in the studied populations.

Unexpectedly, the genetic differentiation between the two localities was low. It can suggest an existing gene flow, despite the Vistula River acting as a geographic barrier. Long distance dispersal is usually underestimated in ecological studies of butterflies as such events are very rare and difficult to follow [65]. However, even a single individual may noticeably contribute to the gene flow between the populations, and genetic studies may be very helpful in revealing such events, for example for *P. alcon* which is considered to be an extremely sedentary species [66]. For *S. orion*, movements of up to 1.5 km are documented [53], which is a much longer distance than the width of the Vistula River. Our molecular analysis could suggest even higher dispersal capabilities, since the studied populations were separated from each other by about 9 km. Additionally our data indicate asymmetry in the gene flow between the sampled sites. This phenomenon could be related to prevailing wind direction in the area and therefore we hypothesize that occasional migration of individuals occurs more frequently from the southwest to the northeast. It may contribute to a better genetic condition of the population located on the east bank of the river.

Another possible explanation of the low genetic differentiation between the studied populations is as follows. At the moment, except for the Parchatka site, only single individuals are observed in scattered localities in the east. In the 1990s, *S. orion* was most numerous in the Skarpa Dobrska Nature Reserve, which is situated on the eastern bank of the river, but much closer to Janowiec (ca 4 km) than to Parchatka (ca 12 km) [17]. Therefore, in the past, the gene flow between the two sides of the river could have been higher than at present. Hence, it cannot be excluded that the revealed pattern of genetic differentiation of *S. orion* population in Poland still reflects historic distribution, when the butterfly used to be much more widespread, to a much greater extent than the present situation, which was strongly influenced by habitat fragmentation. This kind of relationship was also found in other endangered lycaenids, namely *Phengaris arion* [67] and *Pseudophilotes bavius* [68], as well as for *Melitaea cinxia* [69].

This study could contribute to the preparation of an appropriate and effective plan for the species protection in the future. Estimation of an effective population size (*N*e) is often important for determining the conservation status of threatened populations and species [70]. *N*e values for the chequered blue were rather low and similar in both populations. The values seem to be not much lower than the estimated census sizes, but the information provided by the two numbers does not correspond temporally: demographic data were collected in 2015 and 2016, and the samples for genetic analyses in 2018. However, it can be assumed that in 2018, as in the previous years, the P2 population was 2–3 times more numerous than the P1 population. Thus, *N*e/N ratio in both populations should be similar, indicating genetic similarity of the populations and the presence of gene flow between them. Restored or created patches of biotopes need to exist in close proximity to enable gene flow between populations and the spontaneous colonization of new patches of habitat. Finally, even if we assume sufficient gene flow between the populations, we must remember that the total regional population size is very small and very sensitive to genetic deterioration and even extinction, especially taking into consideration seasonal fluctuations in numbers of the Polish populations detected in our studies. Hence, actions dedicated to the protection, improvement, and enlargement of existing habitats are vital to the survival of *S. orion* in the region. Actions undertaken in Finland are a good example [13].

### 4.3. Phylogeographic Pattern

Our results have some implications for the species taxonomy, namely subspecific division. The dominant COI haplotype in Polish populations is the same as that identified in Finland, and it differs only slightly from those found in Norway, Germany, Switzerland, and Romania. Therefore, it may be concluded that there are no phylogenetic grounds for the separation of Nordic subspecies *S. o. subornata* from *S. o. orion.* The morphological differences between both the two forms manifest mostly in colouration of males (they are much bluer in the case of individuals from Fennoscandia). This could be an example of clinal variation as in the case of other lycenids [71,72]. However, the Iberian subspecies *S. o. parvula* seems to be a clearly distinct lineage and its status is certain.

The steppe species do not show a uniform phylogeographic pattern in eastern Central Europe, but they share a unique genetic signature and some distinct units were formed in now isolated steppic habitats [73]. We found three individuals with a rather distant mtCOI haplotype (H2) were found in the population (P1) located on the eastern bank of the Vistula River. The H2 haplotype is close on the tree to H9 and H10 haplotypes detected among individuals from Central Asia (Russia and Kazakhstan). However, it is worth noting that we currently have incomplete genetic data, and filling the geographic gaps across the entire range of the species’ distribution may give a clearer insight into its phylogeographic pattern.

The chequered blue butterfly displays similar but less complex phylogeographic diversity to the Glanville fritillary (*Melitaea cinxia*) populations, which formed some not totally genetically isolated units when compared in north-south and east-south directions. Polish *M. cinxia* population had mixed ancestry as mtCOI haplotypes from Central and Eastern (Siberia and Urals sub-clades) lineages were detected [74]. Other studies performed on butterflies [74,75,76], beetles [77] and mammals [78] show a significant variation in mtDNA markers, which suggests that Poland could be a contact zone for some species, between different phylogenetic lineages. Further studies would allow us to get a more complete picture of the phylogeography of *S. orion*. In Europe, intensive sampling in the southern part of the range is especially important, since this area may be a refugium for genetic diversity of some species, including butterflies [79]. However, peripheral populations, which can be considered Polish populations of *S. orion,* are expected to be generally less diverse [3].

## 5. Conclusions

Mark-release-recapture studies revealed that both studied populations were small, and they fluctuated in numbers, but on the western site, adults were twice as numerous. We found out that genetic variation was largely shared between both sites, but higher for the smaller eastern population. This pattern could be better explained by past distribution, when the butterfly used to be much more widespread on the eastern bank of the river. However, the genetic differentiation between two localities was low. This could suggest an existing gene flow, despite the Vistula River acting as a geographic barrier. We hypothesize that the occasional migration of individuals occurs more frequently from the west to the east, which is consistent with the dominant regional wind direction. It may also contribute to a better genetic condition of the western population. The analysis of available COI sequences of *S. orion* suggests that most of the individuals from the Polish populations are similar to Finish ones, while there are no phylogenetic grounds for the separation of the Nordic subspecies *S. o. subornata* from *S. o. orion,* and the Iberian subspecies *S. o. parvula* is certain. Molecular data suggest an interesting phylogeographic pattern in *S. orion*. However, to gain a complete insight into this pattern, it is important to fill in the gaps in the geographic range of the species with genetic data. Our results can help to create an effective conservation plan for the species in the future.

## Figures and Tables

**Table 1:** Twelve microsatellite loci amplified in 65 individuals of *Scolitantides orion* from the two Polish populations.

Locus	References	Dye	Mix	No. of Cycles	µM	Size Range	*F*null #	
Macu15 *	[30]	6-FAM	SorI (1)	30	0.3	146	ND	ND
Macu44	[31]	VIC	SorI (1)		0.3	180–186	0.013	0.013
boleun01	[32]	NED	SorI (1)		1.8	144–188	−0.191	−0.311
Macu5	[30]	6-FAM	SorII (2)	40	0.3	174–240	−0.057	−0.063
Macu40*	[33]	NED	SorII (2)		0.3	85–167	0.213	0.169
Macu16	[30]	VIC	SorIII (3)	45	0.3	217–303	−0.018	−0.054
Lb4/18 *	[34]	PET	SorIII (3)		0.3	201–287	0.119	0.091
Malc169	[30]	6-FAM	SorIII (3)		0.3	248–332	0.030	0.029
Macu9 *	[30]	NED	SorIII (3)		0.3	181	ND	ND
Macari16 *	[35]	VIC	SorIV (4)	45	0.3	80–228	0.082	0.073
Macari18	[35]	PET	SorIV (4)		0.3	75–157	−0.122	−0.150
Macu29 *	[33]	VIC		45	0.3	91–217	0.190	0.130

Dye—fluorescent dye labeling; Mix—multiplex set (1–4); µM—optimized primer concentration; Size range—observed size ranged of the amplified alleles (in bp); # *F*null were estimated using programs Cervus (left side of the column) and Micro-Checker according to van Oosterhout (right side of the column); ND—not done; * loci excluded from the further analyses.

**Table 2:** Sample sizes and estimated population sizes for the two populations (P1 and P2) of *Scolitantides orion* in Poland in two seasons.

Population	Year	Number of Marked Butterflies		Estimated Population Size *N* ± SE (95% CI)		
		M	F	M	F	All
P1	2015	6	13	8 ± 3 (6–22)	15 ± 3 (13–29)	23 ± 4 (20–39)
	2016	31	20	48 ± 6 (40–63)	37 ± 6 (29–52)	85 ± 8 (66–90)
P2	2015	23	13	40 ± 6 (32–55)	30 ± 6 (22–45)	70 ± 8 (57–90)
	2016	47	27	92 ± 10 (76–116)	72 ± 10 (56–96)	164 ± 14 (138–200)

M—males; F—females. The values in parentheses represent 95% confidence intervals (CI).

**Table 3:** Survival rate and mean lifespan of adult butterflies in two populations (P1 and P2) of *Scolitantides orion* in Poland in two seasons.

Population	Year	Sex	Survival Rate *φ* ± SE (95% CI)	Mean Lifespan (Days) e ± SE (95% CI)
P1	2015	All	0.84 ± 0.04 (0.75–0.91)	5.93 ± 0.11 (3.49–10.35)
	2016	M	0.91 ± 0.04 (0.81–0.96)	10.68 ± 0.04 (4.82–24.49)
	2016	F	0.87 ± 0.06 (0.71–0.95)	7.39 ± 0.06 (2.94–19.99)
P2	2015	M	0.87 ± 0.04 (0.76–0.93)	6.93 ± 0.04 (3.72–13.34)
	2015	F	0.73 ± 0.12 (0.45–0.90)	3.17 ± 0.12 (1.33–9.04)
	2016	All	0.85 ± 0.03 (0.78–0.90)	6.19 ± 0.03 (4.00–9.74)

M—males; F—females. The values in parentheses represent 95% confidence intervals (CI).

**Table 4:** Characterization of six microsatellite loci analysed in 65 individuals of *Scolitantides orion* from two Polish populations (P1 and P2).

Population	All (N = 65)				P1 (N = 34)					P2 (N = 31)				
Locus	A	H_E_	H_O_	PIC	A	H_E_	H_O_	PIC	P_HW_	A	H_E_	H_O_	PIC	P_HW_
Macu44	4	0.54	0.52	0.44	2	0.51	0.53	0.37	1.000	4	0.56	0.52	0.49	0.668
boleun01	6	0.53	0.74	0.46	4	0.54	0.79	0.44	*0.003*	5	0.52	0.68	0.47	0.347
Macu5	12	0.55	0.57	0.52	7	0.58	0.71	0.52	0.271	9	0.51	0.42	0.49	0.198
Macu16	6	0.11	0.11	0.10	5	0.14	0.15	0.14	1.000	3	0.06	0.07	0.06	1.000
Malc169	12	0.67	0.59	0.65	10	0.68	0.58	0.64	*0.015*	8	0.64	0.61	0.60	0.107
Macari18	7	0.61	0.74	0.54	7	0.66	0.77	0.60	0.132	5	0.56	0.71	0.47	0.316
Overall	7.83	0.50	0.55	0.45	5.83	0.52	0.59	0.45		5.67	0.48	0.50	0.43	

N—population size; A—number of alleles; H_E_—expected heterozygosity; H_O_—observed heterozygosity; PIC—mean polymorphic information content; P_HW_—Hardy-Weinberg probability test.

**Table 5:** Molecular diversity indices for mtDNA (COI and ND5) and nuclear (EF-1α and Wgl) genes in two populations (P1 and P2) of *Scolitantides orion* from Poland.

	Nh		h (SE)		π (SE)		S	
Population	P1	P2	P1	P2	P1	P2	P1	P2
COI 632 bp	2	1	0.166 (0.080)	0	0.001 (0.001)	0	4	0
ND5 570 bp	2	2	0.121 (0.075)	0.443 (0.069)	0.0002 (0.0004)	0.0008 (0.0008)	1	1
COI_ND5 1203 bp	3	2	0.234 (0.095)	0.443 (0.069)	0.0005 (0.0005)	0.0004 (0.0004)	5	1
EF-1α 573 bp	4	2	0.118 (0.054)	0.259 (0.065)	0.0004 (0.0005)	0.0005 (0.0006)	4	1
Wgl 338 bp	3	2	0.536 (0.024)	0.474 (0.033)	0.002 (0.002)	0.001 (0.001)	2	1
EF-1α_Wgl 965 bp	5	4	0.568 (0.029)	0.624 (0.034)	0.0008 (0.0007)	0.0008 (0.0006)	6	2

Nh—number of haplotypes; h—gene diversity; *π*—nucleotide diversity; S—number of segregating sites; SE—standard deviation; *F*_ST_, *Φ*_ST_—coefficients of genetic differentiation.

## Supplementary Materials

The following are available online at https://www.mdpi.com/2075-4450/11/9/608/s1 Figure S1: The study species *Scolitantides orion orion*: (a) a male, (b) a female, and (c) a mating pair from Polish populations. Figure S2: The study sites of *Scolitantides orion* in Poland: (a) Parchatka (P1), and (b) Janowiec (P2). Figure S3: Principal Coordinate Analysis based on Nei’s genetic distance matrices between *Scolitantides orion* populations from Poland (P1 population: black diamonds and P2 population: green diamonds). Table S1: Frequencies of 12 haplotypes of the 579 bp COI mitochondrial gene fragment identified in two populations of the chequered blue (*Scolitantides orion*) from Poland (2 haplotypes: H1, H2) and deposited in the GenBank database (11 haplotypes: H1, H3-H12).
