# Supplementary material for: Population Genetic Structure and Demography of the Critically Endangered Chequered Blue Butterfly (Scolitantides orion) in a Highly Isolated Part of Its Distribution Range
Source: Insects. 2020 Sep 8;11(9):608. doi: 10.3390/insects11090608 (PMC7564389; doi:10.3390/insects11090608)
Supplement: Supplementary file 1 [file insects-11-00608-s001.zip › Figure S1.pdf]

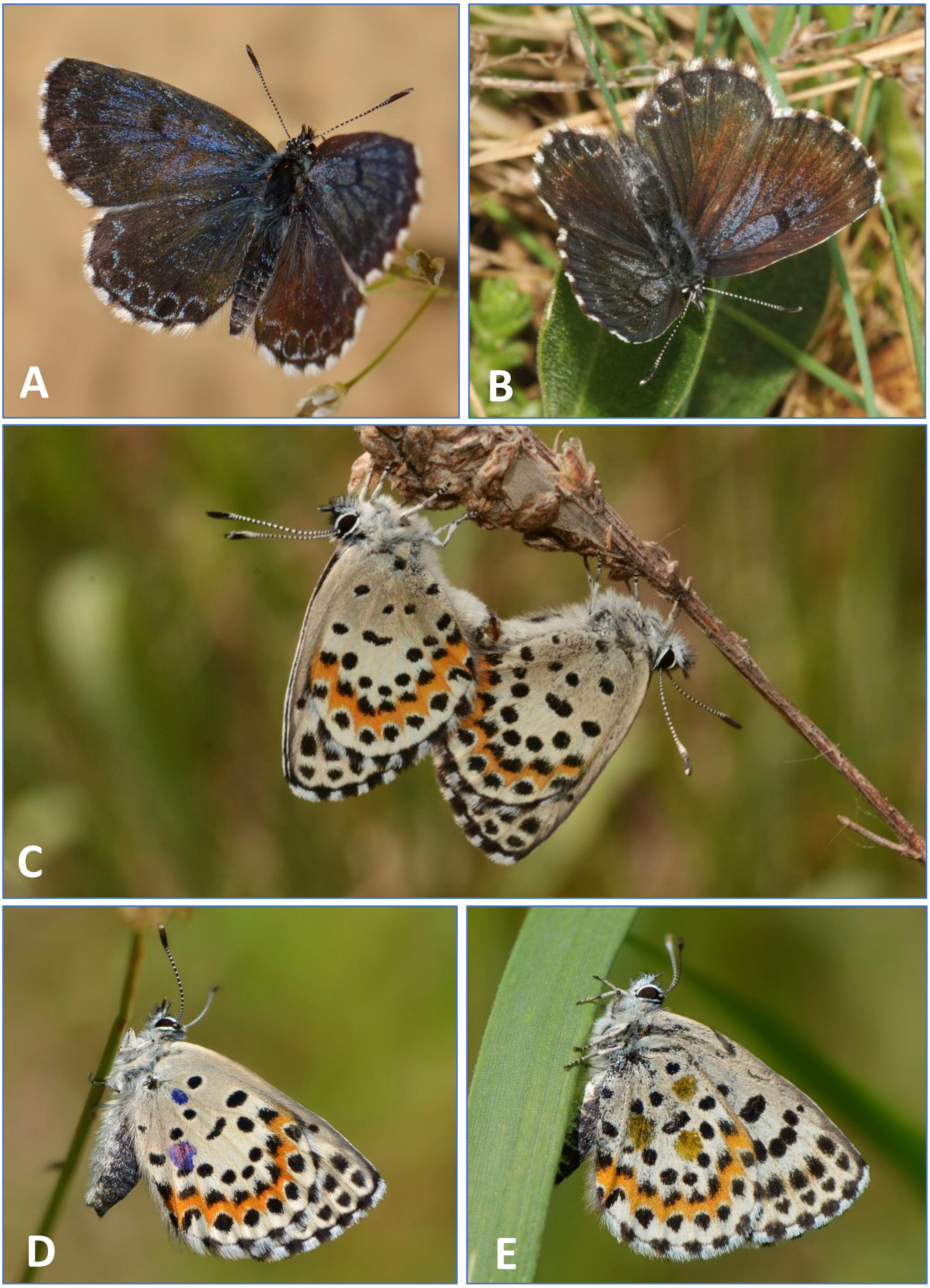

Figure S1: The study species *Scolitantides orion orion:* (A) a male; (B) a female; (C) a mating pair from one of the Polish populations and (D, E) examples of unique identity codes used in the marking system. Photo credits: Łukasz Dawidowicz, Izabela Dziekańska and Marcin Sielezniew.
