# Supplementary material for: Population Genetic Structure and Demography of the Critically Endangered Chequered Blue Butterfly (Scolitantides orion) in a Highly Isolated Part of Its Distribution Range
Source: Insects. 2020 Sep 8;11(9):608. doi: 10.3390/insects11090608 (PMC7564389; doi:10.3390/insects11090608)
Supplement: Supplementary file 1 [file insects-11-00608-s001.zip › Figure S2.pdf]

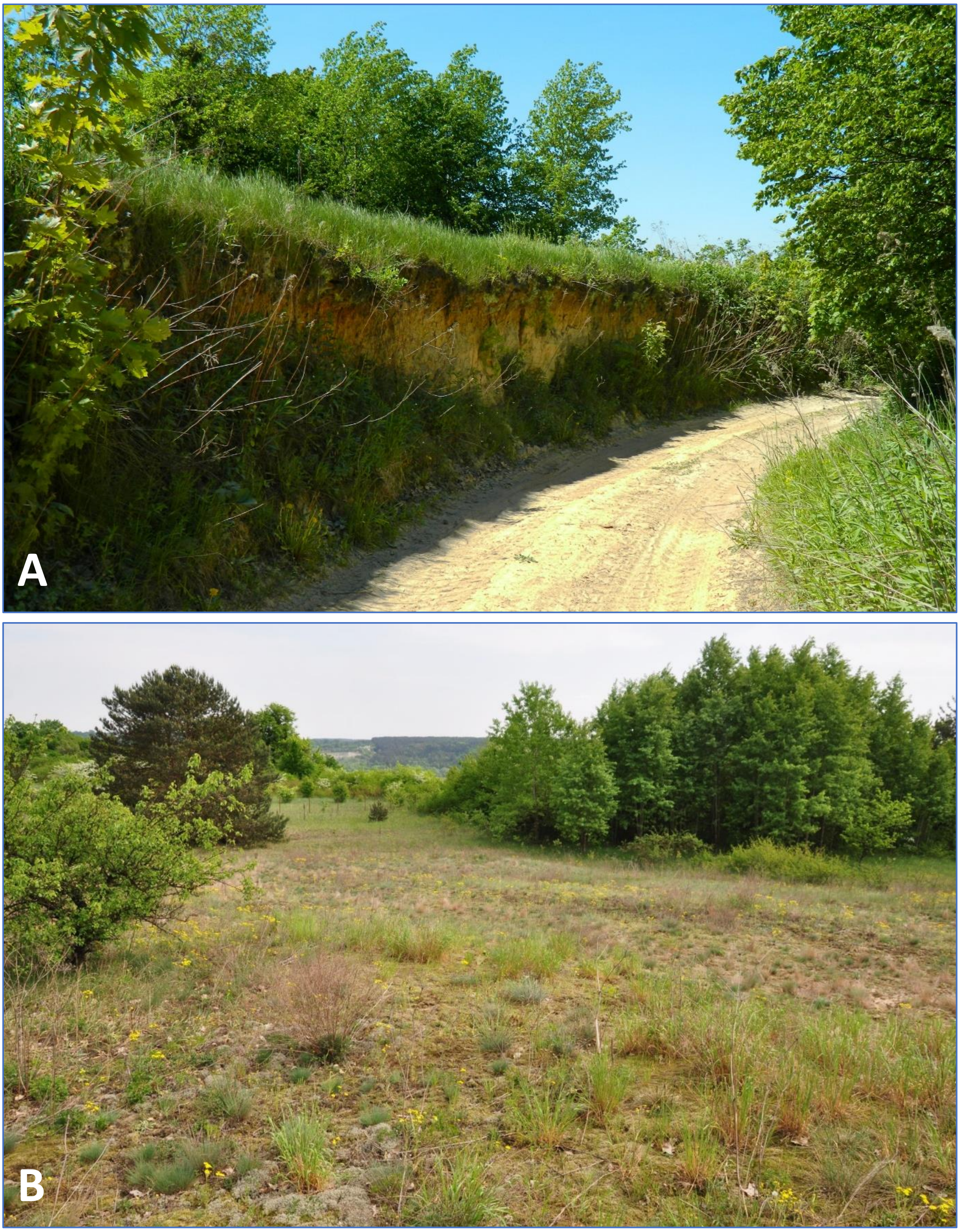

Figure S2: The study sites of *Scolitantides orion* in Poland: (A) Parchatka (P1), and (B) Janowiec (P2). Photo credits: Łukasz Dawidowicz and Marcin Sielezniew.
