# Supplementary material for: Population Genetic Structure and Demography of the Critically Endangered Chequered Blue Butterfly (Scolitantides orion) in a Highly Isolated Part of Its Distribution Range
Source: Insects. 2020 Sep 8;11(9):608. doi: 10.3390/insects11090608 (PMC7564389; doi:10.3390/insects11090608)
Supplement: Supplementary file 1 [file insects-11-00608-s001.zip › Table_S1.pdf]

Table S1. Frequencies of 12 haplotypes of the 579 bp COI mitochondrial gene identified in two populations of the chequered blue butterfly (*Scolitantides orion*) from Poland (2 haplotypes: H1, H2) and deposited in the GenBank database (11 haplotypes: H1, H3-H12).

Population / Haplotype	P1 (N=34)	P2 (N=30)	ES (N=9)	DE (N=2)	CH (N=1)	NO (N=3)	FI (N=1)	RO (N=7)	AL (N=1)	RU (N=1)	KZ (N=2)	KR (N=1)	CN (N=1)
H1	0.91	1.00	0.00	0.00	0.00	0.00	1.00	0.00	0.00	0.00	0.00	0.00	0.00
H2	0.09	0.00	0.00	0.00	0.00	0.00	0.00	0.00	0.00	0.00	0.00	0.00	0.00
H3	0.00	0.00	0.89	0.00	0.00	0.00	0.00	0.00	0.00	0.00	0.00	0.00	0.00
H4	0.00	0.00	0.11	0.00	0.00	0.00	0.00	0.00	0.00	0.00	0.00	0.00	0.00
H5	0.00	0.00	0.00	0.50	0.00	0.00	0.00	0.00	0.00	0.00	0.00	0.00	0.00
H6	0.00	0.00	0.00	0.50	1.00	0.00	0.00	1.00	0.00	0.00	0.00	0.00	0.00
H7	0.00	0.00	0.00	0.00	0.00	1.00	0.00	0.00	0.00	0.00	0.00	0.00	0.00
H8	0.00	0.00	0.00	0.00	0.00	0.00	0.00	0.00	1.00	0.00	0.00	0.00	0.00
H9	0.00	0.00	0.00	0.00	0.00	0.00	0.00	0.00	0.00	1.00	0.50	0.00	0.00
H10	0.00	0.00	0.00	0.00	0.00	0.00	0.00	0.00	0.00	0.00	0.50	0.00	0.00
H11	0.00	0.00	0.00	0.00	0.00	0.00	0.00	0.00	0.00	0.00	0.00	1.00	0.00
H12	0.00	0.00	0.00	0.00	0.00	0.00	0.00	0.00	0.00	0.00	0.00	0.00	1.00

Population symbols (numbers of sequences used from the GenBank database are given in brackets): P1 – Parchatka; P2 – Janowiec; AL – Albania (MH407225); CH – Switzerland (MK186780); CN – China (KT236388); DE – Germany (HM391781, KX044703); ES – Spain (KP870233, KP870366, KP870455, KP870623, KP870781, GU676740, HM901246, HM901388, HM901429); FI – Finland (KT782365); KR – Republic of Korea (GU372588); KZ – Kazakhstan (FJ664037, FJ664038); NO – Norway (KX048470, KX048509, KX048958); RO – Romania (HQ005195, HQ005197, HQ005198, HQ005199, HQ005200, HQ005201, KP870981); RU – Russian Federation (JF854417).
